# Serum Anticholinergic Activity and Cognitive and Functional Adverse Outcomes in Older People: A Systematic Review and Meta-Analysis of the Literature

**DOI:** 10.1371/journal.pone.0151084

**Published:** 2016-03-21

**Authors:** Mohammed Saji Salahudeen, Te-yuan Chyou, Prasad S. Nishtala

**Affiliations:** School of Pharmacy, University of Otago, P O Box 56, Dunedin, 9054, New Zealand; Fatebenefratelli Foundation for Health Research and Education, ITALY

## Abstract

**Introduction:**

Studies have reported associations between serum anticholinergic activity (SAA) and decline in cognitive performance, delirium, and functional impairment. The aim of this meta-analysis was to explore and quantify associations between SAA and adverse cognitive and functional outcomes in older people.

**Materials and Methods:**

A literature search in Ovid MEDLINE, EMBASE, PsycINFO and IPA from 1946–2014 was completed. The primary outcomes of interest were cognitive and functional adverse outcomes associated with SAA in older people aged 55 years and above. The Cochrane Risk-Bias assessment tool was used to assess bias in randomised controlled trials (RCTs). The Newcastle-Ottawa Scale was used to assess the quality of non-RCTs. Meta-analyses were conducted for RCTs and cohort studies separately. Heterogeneity was assessed using *I*^2^ tests.

**Results:**

The primary electronic literature search identified a total of 1559 records in the 4 different databases. On the basis of full-text analysis, 33 studies that met the inclusion criteria. The review included 4 RCTs, 5 prospective cohort studies, 3 longitudinal cohort studies, 17 cross-sectional studies, and 4 case-control studies. Twenty-four of the retrieved studies examined an association between SAA and cognitive outcomes, 2 studies examined an association with SAA and functional outcomes and 8 studies examined associations between SAA and both cognitive, and functional outcomes. The meta-analysis on 4 RCTs showed no association with higher SAA and cognitive performance (*I*^*2*^ = 89.38%, *H*^*2*^ = 25.53 and *p*-value = <0.05) however, the pooled data from 4 observational studies showed elevated SAA was associated with reduced cognitive performance (*I*^*2*^ = 0.00%, *H*^*2*^ = 3.37 and *p*-value = 0.34).

**Conclusion:**

This systematic review summarises the limitations of the SAA on predicting cognitive and functional outcomes in older people. SAA measured by receptor bioassay is flawed and its use in older people with multimorbidity and polypharmacy is questionable.

## Introduction

Medicines with anticholinergic properties are often prescribed to older people for various medical conditions [1, 2]. Anticholinergic burden refers to the cumulative exposure to multiple medicines with anticholinergic properties [3–5]. In early 1980s, a radioreceptor assay, now commonly referred to as serum anticholinergic activity (SAA), was developed by Tune and colleagues to quantify an individual’s overall anticholinergic burden contributed by the cumulative effect of drugs and their metabolites, and potentially by unknown endogenous factors [6, 7]. SAA is generally measured in terms of atropine equivalents (pmol/ml) and ranges from the lowest detectable limit of 0.25 pmol/ml to 25.00 pmol/ml [6, 8–10].

The evidence between association of SAA and adverse outcomes is mixed and mostly derived from case-control or cohort studies. Higher SAA levels have shown to be positively correlated with cognitive impairment in older presurgical patients [11], Alzheimer's patients [12], and in nursing home residents [13, 14]. A study by Chew et al. [8] reported a correlation between SAA and cognitive decline even in moderately to severely demented patients. Rovner et al. reported that higher SAA scores were associated with lower Mini Mental State Examination (MMSE) scores of 24 or less [14]. A community-based cross-sectional study by Mulsant et al. found an association between SAA and decline in MMSE scores with varying degrees of SAA in 90% of the study population [7]. In contrast, Nishtala et al. also found that high SAA medicines were not often associated with neuropsychiatric events [15]. A recent study found no significant difference in SAA levels measured in cerebrospinal fluid and serum of participants with and without delirium [16] A recent cross-sectional study conducted in Finland also found no association between SAA levels and MMSE scores, even though the study reported relatively higher SAA levels [17].

SAA is recognised as a biomarker for cognitive impairment, but concerns whether peripheral SAA measurements predict central nervous system (CNS) effects have been debated [18, 19]. Importantly, no definite threshold level of SAA has been identified that predicts delirium or cognitive dysfunction [20, 21]. There have also been concerns about the variability in SAA bioassay methods, Gerresten and Pollock [19] suggested the use of human cloned selective muscarinic receptor subtypes to improve the specificity and reliability of the bioassay for predicting CNS effects. SAA reflects the state in the peripheral blood and is not necessarily associated with conditions in the central nervous system [22]. Literature shows only limited studies are available on finding associations between SAA and functional outcomes [23, 17] and there is a lack of systematic review that has been identified in this area. Functional impairment may be caused by peripheral adverse effects like accommodation difficulties, tachycardia and gait disturbance [24]. Hence, in this review we have included and assessed the functional outcomes such as activity of daily living, physical function, and psychomotor function to answer the existing gap.

### Objectives

The validity of Serum Anticholinergic Activity (SAA) as a biomarker for cognitive and functional impairment in older people is a subject of incessant debate [20, 21, 25]. Therefore, a systematic review is needed to appraise and summarise the current evidence regarding SAA and associations with adverse cognitive and functional outcomes such as change in cognition, delirium, and activities of daily living in older people.

We therefore conducted (1) a systematic review of published studies of randomised and non-randomised controlled trials that assessed the association between SAA and adverse outcomes in older people; and (2) a meta-analysis to quantify the association between elevated SAA and its impact on cognition.

## Materials and Methods

### Data sources and search strategy

A literature search in Ovid MEDLINE, EMBASE, PsycINFO and International Pharmaceutical Abstracts (IPA) covering the period 1946—September 2014 was completed to identify SAA and adverse outcomes in older people using the keywords; (anticholinergic*.mp), AND (cogniti#.mp) AND ("aged/ or elder*.mp. or frail.mp. or geriatric*.mp. or seniors.mp. or “old#.mp "). The search was then limited to English language AND humans. The MEDLINE search strategy is presented in S1 Table.

The Preferred Reporting Items for Systematic Reviews and Meta-Analyses (PRISMA) criteria was employed to report this systematic review and meta-analyses [26]. A protocol was not registered and ethics review was not required for conducting this study. A PRISMA checklist for systematic review is depicted as supplementary information.

Following the primary systematic search to identify relevant studies, the reference list from each study was searched to identify potentially relevant articles examining the association between SAA and adverse outcomes. Web of Science and Google Scholar were used to track prospective citing of references of selected articles. Potentially relevant articles identified were then reviewed according to the predefined inclusion and exclusion criteria.

**Fig 1:**
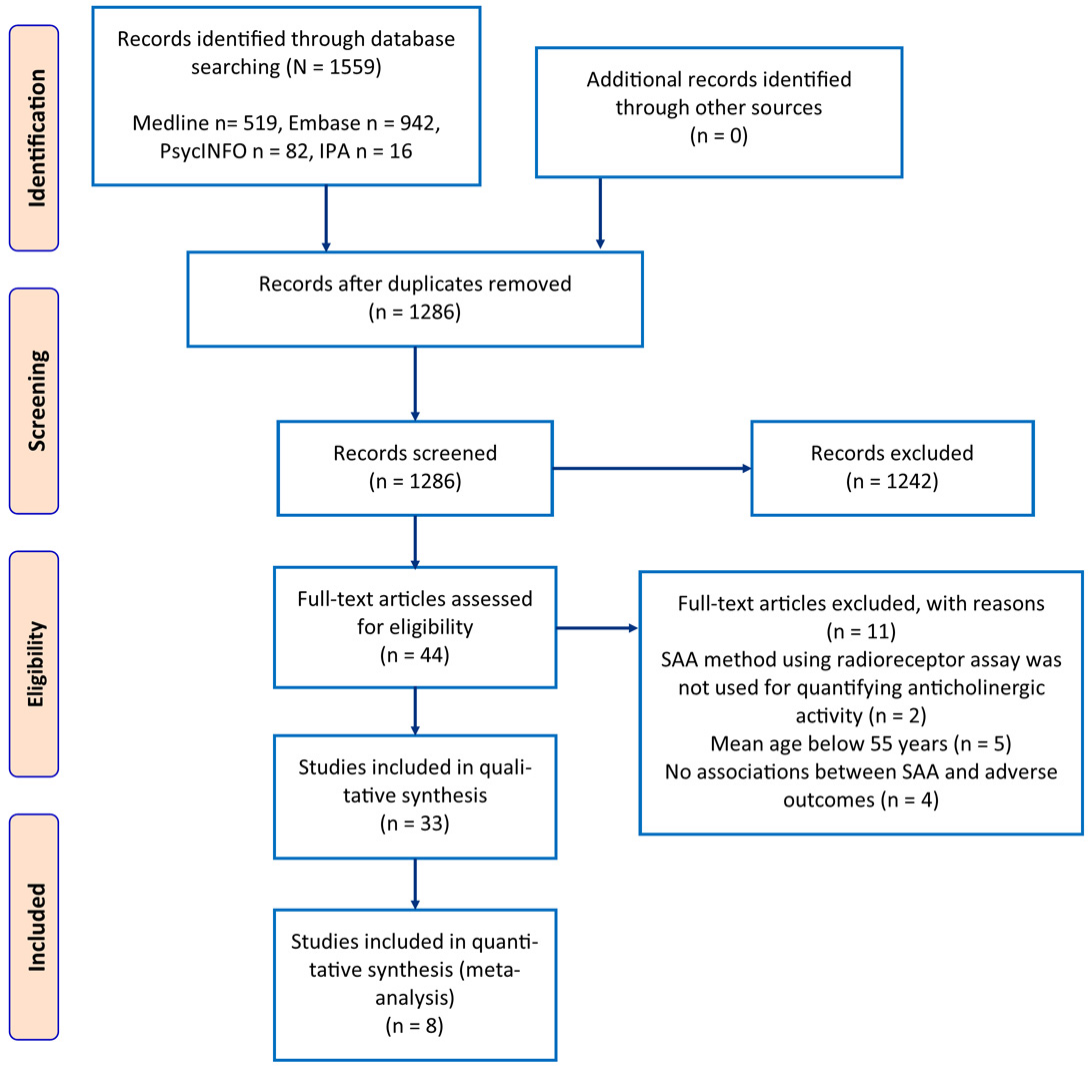
PRISMA flow diagram of study selection process.

A PRISMA flowchart of study selection process is depicted in Fig 1.

### Study screening and selection

The title and abstract of the publication were screened by two independent reviewers for its eligibility for inclusion in the review process (M.S.S. and P.S.N.). The eligible studies were subject to a thorough full text analysis for relevance and pre-defined inclusion criteria. Studies that met the following criteria were included in the final review.

1. Studies that include participants of either sex, of mean age 55 years or older and living in community or primary care or nursing homes or hospital settings.
2. Studies that reported the use of SAA as quantification method either using a radioreceptor assay technique or *in vitro* measurement of individual muscarinic receptor activity.
3. Studies that included a tool to assess cognitive or/and functional outcomes.
4. Any intervention that employed SAA as a quantification tool to measure adverse clinical outcomes.

We excluded articles in languages other than English, as well as case reports, commentaries, letters and editorials from the primary search and citation analysis. Anticholinergic rating scales based predominantly on SAA were also excluded from the review.

Eleven studies [27–37] were excluded from the analysis as they failed to meet the inclusion criteria. The studies excluded considered age less than 55 years (n = 5), where SAA was not the primary method employed to quantify anticholinergic burden (n = 2) and where adverse outcomes were not examined (n = 4). A detailed summary of excluded studies is depicted in S2 Table.

The primary aim of this review was to evaluate cognitive and functional adverse outcomes such as change in cognition, delirium, and activities of daily living associated with SAA in older people. In this study, association between delirium and SAA was also assessed as a primary outcome measure.

### Risk of bias assessment

The quality of the included studies was critically appraised by two authors (M.S.S. and P.S.N.). The Cochrane Risk of Bias tool [38] was used to assess the methodological quality of RCTs. The Newcastle-Ottawa scale [39] which consists of three broad criterions on selection, comparability and study outcome (cohort studies) or based on exposure (case-control studies) was used to assess the quality of the non-RCTs. Differences between review authors concerning eligibility were reviewed by the third author (T.Y.C.) and decisions were made by consensus.

### Data extraction and synthesis

Two reviewers (M.S.S. and P.S.N.) compiled data onto standardised format based on study population, study design, sample size, study duration, mean age, mean SAA and adverse outcome measures. The primary outcomes of interest were cognitive and functional adverse outcomes including change in cognition, delirium, and activities of daily living associated with SAA quantified by using radioreceptor assay or *in vitro* muscarinic receptor activity assay.

A citation analysis was performed to identify and compare the clinical utility of SAA and to evaluate its association with adverse outcomes in older people. Studies that used the SAA for assessing the adverse outcomes in older people aged 55 years and above are reported in this review.

### Statistical analysis

For meta-analysis, the required standard deviations (SD) and mean values were extracted from the included studies. We contacted the authors for information that were not shown or derivable from the original publication. From the extracted study information, statistical analysis was pooled for doing a meta-analysis, if there were minimum three studies assessing the same outcome measure.

The data was meta-analysed using the package METAFOR in R 3.1.2. The data from 3 RCT studies were pooled to quantify the impact of SAA on cognitive outcomes. A separate meta-analysis was completed for observational studies that reported the same outcome measures. The primary outcome for measure of cognitive performance was change in MMSE scores. The means and standard deviations (SD) of MMSE scores in the intervention and control groups were converted into a standardised effect size. A random effects model was used to combine the standardised effect sizes with 95% confidence interval. Heterogeneity was assessed using *I*^2^ statistics. A statistically significant *I*^2^ suggests that variation of standardised effect sizes among the included studies is due to the uniqueness of each study (i.e. a significant heterogeneity) rather than random variation.

Funnel plot (scatterplot of the intervention effect estimate of individual studies against outcome measure of each study size, is a visual aid for detecting bias or systematic heterogeneity) was used to identify studies that were potential outliers and over-presented in the random effect modelling. All data were distributed symmetrically in the funnel plot and therefore, publication bias was not evident. MMSE scores outside the funnel-shaped region were excluded, and the combined standardised effect size was recalculated without the influential data by random effect modelling. Studies were also excluded if the MMSE scores were not reported as means and SD, or information provided was incomplete.

## Results

### Search results

The primary search using four databases identified a total of 33 studies as being relevant to this systematic review. A qualitative description of the included studies is shown in Table 1.

**Table 1:** Qualitative summary of included study characteristics between serum anticholinergic activity and cognitive and functional outcomes. SAA = Serum Anticholinergic Activity; MMSE = Mini Mental State Examination; IQCODE = Informant questionnaire on Cognitive Decline in the Elderly; SPMSQ = Short Portable Mental Status Questionnaire; SIB = Severe Impairment Battery; CAM = Confusion Assessment Method; BI = Barthel Index; AD = Alzheimer’s disease; FAST = Functional Assessment Staging; BEHAVE-AD = Behavioural Pathology in Alzheimer’s Disease Rating Scale; BARS = Brief Agitation Rating Scale; POCD = Postoperative cognitive decline; CAM-ICU = Confusion Assessment Method for critically ill patients in Intensive Care Unit; MCI = Mild Cognitive Impairment; DI = Delirium Index; SDM = Symbol Digit Modalities; SDC = Saskatoon Delirium Checklist; IQR = Interquartile range; PGDRS = Psychogeriatric Dependency Rating Scale; DRS = Dementia Rating Scale; WMH = White Matter Hyperintensities; ICU = Intensive care unit; RCT = Randomised Controlled Trial; ADL = Activities of Daily Living; IADL = Instrumental Activities of Daily Living; CERAD = Consortium to Establish a Registry for Alzheimer Disease; GDS = Geriatric Depression Scale; SD = Standard Deviation; DSM = Diagnostic and Statistical Manual of Mental Disorders.

Studies used SAA	Study design	Study setting / participants	Mean (SD) age (years)	Study duration	Adverse outcome(s) studied	Significant association
**RCT**						
**Kersten et al., Norway 2013 [41]**	RCT, single-blinded	nursing home residents with AD, N = 87	86.0 ± 5.6	8 weeks	mouth dryness (whole-mouth resting saliva flow)	–
					cognitive function (MMSE, CERAD)	–
**Lackner et al., USA 2008 [40]**	RCT, double-blinded	nursing home, N = 50	88.6 ± 6.2	4 weeks	cognitive functions, (MMSE, SIB)	–
					delirium (CAM, BARS)	–
**Miller et al., Canada 1988 [11]**	RCT, double blinded	inpatients, N = 36	67.0 ± 5.9	unclear	cognitive performance; MMSE	–
					SDM	–
					SDC (delirium)	+
					Rey Auditory-verbal learning test	+
**Tollefson et al., USA 1991 [13]**	RCT, parallel-arm	nursing home, N = 34	79.0 ± 9.7	4 weeks	cognitive performance (MMSE)	–
**Longitudinal cohort**						
**Golinger et al., USA 1987 [46]**	Longitudinal cohort	surgical ICU, N = 25	58.1 (29–76)	3 months	Delirium (DSM)	+
**Kashyap et al., Canada 2014 [48]**	Longitudinal cohort	outpatient clinic, N = 121	71 ± 7.2	1-year	change in cognition	–
**van Munster et al., Netherlands 2012 [47]**	Longitudinal study	hospitalised pts, N = 142	83.9 ± 6.9	3 ½ years	delirium (CAM)	–
**Cross-sectional**						
**Chew et al., USA 2005 [8]**	Cross-sectional	geropsychiatric ward, N = 26 (demented pts)	83.6 ± 5.8	2 year and 2 months	cognitive function (MMSE)	+
					SIB	–
**Flacker et al., USA 1998 [51]**	Cross-sectional	medical unit, N = 67	85.5 ± 6.1	3 ½ months	ADL	–
**Flacker et al., USA 1999 [52]**	Cross-sectional	nursing home, N = 22	88.0 ± 4.5	1 year	cognitive performance scale	+
					SAA level for acute illness	+
**Hori et al., Japan 2011 [57]**	Cross-sectional	hospital visited AD patients, N = 76	74.3 ± 8.1	1 year 11 months	cognitive and psychiatric symptoms; MMSE, BEHAVE-AD	+
					FAST	+
**Kersten et al., Norway 2013 [58]**	Cross-sectional	nursing home residents, N = 87	86.0 ± 5.6	1 year	mouth dryness	+
					MMSE, CERAD	–
					functional outcome (ADL)	–
**Konishi et al., Japan 2010 [55]**	Cross-sectional	psychogeriatric inpatients with AD, N = 76	78.9 ± 7.2 (SAA > 1.95 nmol); 77.9 ± 7.1 (SAA ≤1.95 nmol)	1 year and 10 months	cognitive functions (MMSE)	+
					FAST score	+
**Lampela et al., Finland 2013 [17]**	Cross-sectional	community-dwelling, N = 621	81.7 ± 4.9	3 years	adverse events	–
					cognitive function (MMSE, GDS)	–
					functional outcomes (ADL, IADL)	–
**Mangoni et al., Netherlands 2013 [59]**	Cross-sectional	hospitalised patients with hip fracture, N = 71	84 ± 6	3 ½ years	cognitive impairment (IQCODE/ MMSE)	+
					physical function (Katz ADL)	+
**Mulsant et al., USA 2003 [7]**	Cross-sectional	community patients, N = 201	78.2 ± 5.2	2 ½ years	cognitive performance (MMSE)	+
**Mussi et al., Italy 1999 [53]**	Cross-sectional	geriatric inpatients, N = 61	79.2 ± 11.6	2 months	delirium (CAM) with elevated SAA	+
**Nebes et al., USA 1997 [50]**	Cross-sectional	geropsychiatric ward, N = 36	69 (6)	1 year	cognitive performance; DRS	–
					total immediate recall	–
					delayed recall	+
					percent retention	+
**Nebes et al., USA 2005 [54]**	Cross-sectional	community-based, N = 134	73.3 ± 3.8	not mentioned in the study	cognitive decrements based on WMH volume	+
**Nebes et al., USA 2007 [23]**	Cross-sectional	community-dwelling, N = 90	72.1 ± 4.1	not mentioned in the study	functional outcomes; psychomotor function (gait speed, simple response time) to predict falls	+
**Nebes et al., USA 2011 [56]**	Cross-sectional	community-based, N = 152	72.0 ± 4	not mentioned in the study	cognitive function, processing-speed	+
					working-memory	–
**Rovner et al., USA 1988 [14]**	Cross-sectional	demented nursing home patients, N = 22	80.8 ± 9.6	unclear	MMSE	–
					self-care capacity (PGDRS)	+
**Thomas et al., Germany 2008 [18]**	Cross-sectional	hospitalised pts, N = 61	86.2 ± 4.5	7 months	delirium (DI), MMSE, IQCODE, SPMSQ	+
					functional outcome (BI)	+
**Tune et al., USA 1993 [49]**	Cross-sectional	surgical ICU pts, N = 25	58.1	3 weeks	delirium (DSM)	+
**Case-control**						
**Mach Jr et al., USA 1995 [10]**	Case-control	hospital medical ward, N = 22	71.3 ± 7.0	1-year	delirium (DSM)	+
**Plaschke et al., Germany 2010 [60]**	Case-control	surgical patients, N = 30	64.5 ± 13	8 months	cognitive function (neuropsychological tests)	–
**Plaschke et al., Germany 2013 [61]**	Case Control	hospitalised pts, N = 117	69.3 ± 8.0	1 year and 3 months	cognitive function (neuropsychometric testing), IQCODE	–
					subjective memory complaints	–
**Thienhaus et al., USA 1990 [12]**	Case-control	geropsychiatric inpatients, N = 28	65 (9)	unclear	cognitive performance (MMSE, Digit Retention Span, word recognition category retrieval, Self-rated Memory Scale)	–
**Prospective cohort**						
**Plaschke et al., Germany 2007 [44]**	Prospective, cohort	intensive care unit patients, N = 37	63.6 ± 11.6	5 months	delirium using electroencephalographic data (CAM-ICU)	–
**Remillard et al., Canada 1994 [43]**	Prospective cohort	nursing home, N = 31	85.1 ± 7.0	unclear	MMSE	–
					SDM	+
**Rossi et al., Switzerland 2014 [45]**	Prospective cohort	surgical inpatients, N = 70	72 (67–77)	1 week	cognitive function (MMSE, CERAD)	–
**Tune et al., USA 1981 [42]**	Prospective cohort	postcardiotomy patients, N = 29	55	2 weeks	delirium (MMSE)	+
**Watne et al., Norway & UK 2014 [16]**	Prospective cohort	from 2 hospitals, N = 148; n = 52, hospital 1 (Edinburgh)	84.0 (median)	1 year and 8 months	IQCODE	+
					Katz ADL	–
		n = 96, hospital 2 (Oslo)	84.0 (median)	1 year and 8 months	IQCODE	+
					Barthel ADL	+

The primary electronic literature search identified a total of 1559 articles from 4 different databases such as Ovid MEDLINE, EMBASE, PsycINFO, and IPA. EndNote was used to eliminate duplicates and we considered 1286 articles for screening. Out of 1286 screened articles based on title and abstract, only 44 were eligible for full-text analysis. From the eligible 44 studies, 11 were excluded on full text analysis according to the set inclusion and exclusion criteria. Hence, in total, 33 studies were included in this review that considered SAA in the estimation of anticholinergic burden. The complete study selection process is portrayed in Fig 1.

### Summary of study findings

The 33 studies retrieved from 1981 through 2014 comprised of 4 RCTs (12%) [11, 13, 40, 41], 5 prospective cohort studies (15%) [16, 42–45], 3 longitudinal cohorts (9%) [46–48], 17 cross-sectional studies (52%) [7, 8, 14, 17, 18, 23, 49–59], and 4 case-control studies (12%) [10, 12, 60, 61] that validated SAA and association with adverse outcomes.

From the aforementioned studies, majority examined (n = 24) an association between SAA and cognitive outcomes [7, 8, 10–13, 40–50, 52–54, 56, 57, 60, 61] using mostly MMSE as a standard measure of cognitive performance. Limited studies (n = 8) reported an association between SAA and both cognitive, and functional outcomes [14, 16–18, 55, 57–59]. Only 2 studies [62, 63] examined an association between SAA and functional outcomes. Combined, twenty-seven per cent (n = 10) of the studies included in this review showed an association between elevated SAA and functional limitations in Activities of Daily Living (ADL) [16, 51, 58, 59], Barthel Index (BI) [18] and Functional Assessment Staging (FAST) [55, 57].

The study settings varied, and included hospitalised patients (n = 19, 58%), nursing home residents (n = 7, 21%), community people (n = 5, 15%) and ambulatory care patients (n = 2, 6%). Thirty per cent (n = 10) of included studies showed an association between SAA and delirium. In these studies, delirium was assessed using several tools and a meta-analysis was not undertaken due to heterogeneity of the study designs, diversity in the interventions and differences in outcome measures. Summary of the heterogeneity of the included studies between SAA and delirium is depicted in S3 Table. Limited studies examined SAA and functional outcomes and in light of heterogeneity, a meta-analysis was not undertaken instead, a descriptive approach was commenced due to methodological limitations. Overall, the studies included in this review had a variable study duration ranging from one week up to three and half years. Study characteristics describing SAA and association with adverse outcomes in older people are shown in Tables 1 and 2.

**Table 2:** Quantitative summary of included study characteristics between serum anticholinergic activity and cognitive and functional outcomes. SAA = Serum Anticholinergic Activity; MMSE = Mini Mental State Examination; IQCODE = Informant questionnaire on Cognitive Decline in the Elderly; SPMSQ = Short Portable Mental Status Questionnaire; SIB = Severe Impairment Battery; CAM = Confusion Assessment Method; BI = Barthel Index; AD = Alzheimer’s disease; FAST = Functional Assessment Staging; BEHAVE-AD = Behavioural Pathology in Alzheimer’s Disease Rating Scale; BARS = Brief Agitation Rating Scale; POCD = Postoperative cognitive decline; CAM-ICU = Confusion Assessment Method for critically ill patients in Intensive Care Unit; MCI = Mild Cognitive Impairment; DI = Delirium Index; SDM = Symbol Digit Modalities; IQR = Interquartile range; PGDRS = Psychogeriatric Dependency Rating Scale; DRS = Dementia Rating Scale; WMH = White Matter Hyperintensities; ICU = Intensive care unit; RCT = Randomised Controlled Trial; ADL = Activities of Daily Living; IADL = Instrumental Activities of Daily Living; CERAD = Consortium to Establish a Registry for Alzheimer Disease; GDS = Geriatric Depression Scale; CI = Cognitive impairment; MANCOVA = Multivariate Analysis of Covariance; ANOVA = Analysis of Variance; ANCOVA = Analysis of Covariance; N.S = Not Significant; pmol = Picomol

Studies used SAA	Study design	Outcome measure	Sample size (n)		Mean SAA (pmol/mL)		Outcome of interest		Statistical test	*p* value
			Control group	Intervention group	Control/ Pre	Intervention/ Post	Control/pre	Intervention/post		
**RCTs**										
**Kersten et al., Norway 2013 [41]**	RCT, single-blinded	CERAD	31	37	3.80 (2.29–8.0) (median), (n = 26)	4.27 (2.19–6.39) (median), (n = 35)	13.06 ± 6.26	14.46 ± 5.92	ANCOVA	.48
		MMSE	30	34	3.80 (2.29–8.0) (median), (n = 26)	4.27 (2.19–6.39) (median), (n = 35)	19.7 ± 5.21	20.68 ± 5.18	ANCOVA	.57
		saliva flow (g/min)	27	34	3.80 (2.29–8.0) (median), (n = 26)	4.27 (2.19–6.39) (median), (n = 35)	0.27 (0.16–0.49)	0.25 (0.09–0.60)	ANCOVA	.34
**Lackner et al., USA 2008 [40]**	RCT, double-blinded	MMSE	24	26	1.15 (0.0–5.05) (median), (n = 24)	0.95 (0.0–6.20) (median), (n = 26)	13.7 ± 0.9	15.2 ± 0.8	Correlation analysis	.25
		SIB	24	26	1.15 (0.0–5.05) (median), (n = 24)	0.95 (0.0–6.20) (median), (n = 26)	87.0 ± 1.6	87.1 ± 1.6	Correlation analysis	.72
		CAM	24	26	1.15 (0.0–5.05) (median), (n = 24)	0.95 (0.0–6.20) (median), (n = 26)	1.8 ± 0.3	2.0 ± 0.3	Correlation analysis	.96
		BARS	24	26	1.15 (0.0–5.05) (median), (n = 24)	0.95 (0.0–6.20) (median), (n = 26)	16.1 ± 1.8	16.9 ± 1.9	Correlation analysis	.75
**Miller et al., Canada 1988 [11]**	RCT, double blinded	MMSE	16	16	11.6 ± 18.2 (n = 16)	121.1 ± 85.5 (n = 14)	28.3 ± 2.2	26.7 ± 3.5	ANCOVA	N.S
		SDM	17	16	11.6 ± 18.2 (n = 16)	121.1 ± 85.5 (n = 14)	36 ± 12.1	28.4 ±13.3	ANCOVA	N.S
		Delirium	17	16	11.6 ± 18.2 (n = 16)	121.1 ± 85.5 (n = 14)	37 ± 2.7	33.3 ± 4.1	ANCOVA	.02
		Rey audio-verbal learning test	16	16	11.6 ± 18.2 (n = 16)	121.1 ± 85.5 (n = 14)	4.4 ± 0.7	3.2 ± 2.0	ANCOVA	< .01
**Tollefson et al., USA 1991 [13]**	RCT, parallel-arm	MMSE	19	15	2.49 ± 3.9	1.89 ± 3.4	22.59 ± 4.76	23.03 ± 4.92	Correlation analysis	< .01
**Longitudinal cohort**										
**Golinger et al., USA 1987 [46]**	Longitudinal cohort	drug-risk number for delirium	16	9	0.81 ± 1.0 (delirium)	4.67 ± 3.3 (no delirium)	12.0 ± 9.2 (delirium)	8.3 ± 6.4 (no delirium)	t-test	.3 (N.S)
**Kashyap et al., Canada 2014 [48]**	Longitudinal cohort	change in cognition (SAA)	68	21, 20, 12	1.03 ± 0.75	0.08 ± 1.2	0.83 (median)	0.65, 0.76, 0.50 (median)	Kruskal-Wallis one-way ANOVA	.87
**van Munster et al., Netherlands 2012 [47]**	Longitudinal study	effect of delirium onset on SAA level as effect size, adjusted for CI	70	72	3.4	4.2	28.8 (SD not reported)		Mixed-model regression	< .05
**Cross-sectional**										
**Chew et al., USA 2005 [8]**	Cross-sectional	Correlation study; MMSE and SAA	25 in total		1.06 ± 1.2 (overall)		r = -0.398		Spearman correlation	.049
		SIB and SAA	28 in total				r = -0.405		Spearman correlation	095
**Flacker et al., USA 1998 [51]**	Cross-sectional	ADL	47	20	0.7 ± 0.8	1.8 ± 1.6	1.5 ± 2.2	4.0 ± 2.6	t-test	< .001
**Flacker et al., USA 1999 [52]**	Cross-sectional	changes in SAA level in individuals with febrile illness between study entry and at one-month follow-up	14	8	0.65 ± 0.51 (entry, delirium)	0.69 ± 0.85 (entry, no delirium)	0.08 ± 0.12 (follow-up, delirium)	0.10 ± 0.16 (follow-up, no delirium)	ANOVA	< .01 for overall change
**Hori et al., Japan 2011 [57]**	Cross-sectional	MMSE	50	26	<1.95	4.14 ± 2.70	13.16 ± 8.27	8.89 ± 8.40	Student t-test	.0367
		FAST	50	26	<1.95	4.14 ± 2.70	4.78 ± 0.98	5.46 ± 1.21	Student t-test	.0096
		BEHAVE-AD: delusion	50	26	<1.95	4.14 ± 2.70	1.2 ± 1.7	3.4 ± 1.3	Student t-test	< .0001
		BEHAVE-AD: hallucination	50	26	<1.95	4.14 ± 2.70	0.7 ± 1.0	1.9 ± 1.0	Student t-test	< .0001
		BEHAVE-AD: activity disturbance	50	26	<1.95	4.14 ± 2.70	2.1 ± 2.2	2.3 ± 2.2	Student t-test	.7162
		BEHAVE-AD: aggressiveness	50	26	<1.95	4.14 ± 2.70	1.1 ± 1.7	1.9 ± 2.1	Student t-test	.0714
		BEHAVE-AD: rhythm disturbance	50	26	<1.95	4.14 ± 2.70	0.6 ± 0.8	1.7 ± 0.7	Student t-test	< .0001
		BEHAVE-AD: affection	50	26	<1.95	4.14 ± 2.70	1.0 ± 1.2	0.6 ± 0.8	Student t-test	.1590
		BEHAVE-AD: anxiety	50	26	<1.95	4.14 ± 2.70	1.4 ± 1.8	1.7 ± 1.8	Student t-test	.6278
**Kersten et al., Norway 2013 [58]**	Cross-sectional	MMSE	72 (EM, extensive metaboliser)	8 (PM, poor metaboliser)	4.2 (2.4, 7.0) for EM	10.3 (5.7, 39.9) for PM	19.5 (17,22)	24 (16,25.5)	Mann-Whitney test	N.S
		CERAD	72 (EM, extensive metaboliser)	8 (PM, poor metaboliser)	4.2 (2.4, 7.0) for EM	10.3 (5.7, 39.9) for PM	12 (9,14)	9.5 (7.25,14.3)	Mann-Whitney test	N.S
		ADL	72 (EM, extensive metaboliser)	8 (PM, poor metaboliser)	4.2 (2.4, 7.0) for EM	10.3 (5.7, 39.9) for PM	4 (3,5)	4 (3,5)	Mann-Whitney test	N.S
		mouth dryness (whole mouth resting salivary flow)	72 (EM, extensive metaboliser)	8 (PM, poor metaboliser)	4.2 (2.4, 7.0) for EM	10.3 (5.7, 39.9) for PM	0.7 (0.4,1.2) (EM)	1.34 (0.1,2.3) (PM)	Mann-Whitney test	N.S
**Konishi et al., Japan 2010 [55]**	Cross-sectional	MMSE	50	26	<1.95	4.14 ± 2.7	13.16 ± 8.27	8.89 ± 8.40	t-test	.0367
		FAST	50	26	<1.95	4.14 ± 2.7	4.78 ± 0.98	5.46 ± 1.21	t-test	.0096
**Lampela et al., Finland 2013 [17]**	Cross-sectional	MMSE, GDS, ADL, IADL, short distance vision	12/609 with and without dementia		median 9.3 (2.27–82.7) Overall		numerical data not shown		Kruskall-Wallis one-way ANOVA	N.S
**Mangoni et al., Netherlands 2013 [59]**	Cross-sectional	Regression analysis: SAA vs ADL	71 in total		median 2.8 (Range 1.1–4.9)		β = 0.39		Linear regression and Cox regression	0.001
		3-month mortality vs SAA	71 in total		median 2.8 (Range 1.1–4.9)		HR = 0.07		Linear regression and Cox regression	0.07
		1-year mortality vs SAA	71 in total		median 2.8 (Range 1.1–4.9)		HR = 1.10		Linear regression and Cox regression	0.11
**Mulsant et al., USA 2003 [7]**	Cross-sectional	MMSE (≤24)	n = 21 with undetectable SAA	n = 159 with low SAA; n = 21 with high SAA	<0.25 (undetectable)	0.25–2.79 (detectable); ≥2.80 (high)	4.8%	7.6%; 28.6%	Pearson χ^2^ test	.006
**Mussi et al., Italy 1999 [53]**	Cross-sectional	SAA level for delirious and non-delirious individuals	49	12	3.9 ± 8.4	23.0 ± 15.5	3.9 ± 8.4	23.0 ± 15.5	t-test	< .004
**Nebes et al., USA 1997 [50]**	Cross-sectional	DRS	17	19	0.0	0.28 ± 0.26	138.6 ± 3.0	135.6 ± 3.6	ANOVA	N.S
		total immediate recall	17	19	0.0	0.28 ± 0.26	26.1 ± 7.2	21.9 ± 7.1	ANOVA	.24
		delayed recall	17	19	0.0	0.28 ± 0.26	6.5 ± 2.5	4.2 ± 2.5	ANOVA	< .05
		percent retention	17	19	0.0	0.28 ± 0.26	86% ± 35%	58% ± 25%	ANOVA	< .05
**Nebes et al., USA 2005 [54]**	Cross-sectional	cognitive decrements based on WMH volume	n = 35, no SAA	n = 69, moderate; SAA; n = 30, high SAA	0.0	1.96 ± 1.0; 6.1 ± 1.7	6.2 ± 7.7	6.3 ± 11.9; 9.2 ± 15.2	ANOVA	< .005 (high SAA group correlated with WHM volume)
**Nebes et al., USA 2007 [23]**	Cross-sectional	gait speed	n = 29, low SAA	n = 33, medium SAA; n = 26, high SAA	0.36 ± 0.34	1.36 ± 0.31	4.32 ± 0.78	4.77 ± 1.06; 5.08 ± 0.88	MANCOVA	.0109
		simple response time	n = 29, low SAA	n = 33, medium SAA; n = 26, high SAA	0.36 ± 0.34	3.42 ± 2.33	244.4 ± 40.0	276.4 ± 56.2; 285.7 ± 63.4	MANCOVA	.0078
**Nebes et al., USA 2011 [56]**	Cross-sectional	processing-speed (n = 75); perceptual comparison	n = 76, low-paraxanthine	n = 76, high-paraxanthine	1.72 ± 2.03	1.35 ± 1.37	781.4 ± 158.9	755.2 ± 133.5	Pearson correlation	< .009
		processing-speed (n = 75); conceptual comparison	n = 76, low-paraxanthine	n = 76, high-paraxanthine	1.72 ± 2.03	1.35 ± 1.37	799.5 ± 147.3	795.6 ± 134.1	Pearson correlation	< .017
		processing-speed (n = 75); working-memory (N Back)	n = 76, low-paraxanthine	n = 76, high-paraxanthine	1.72 ± 2.03	1.35 ± 1.37	32.6 ± 13.5	34.1 ± 12.6	Pearson correlation	N.S
**Rovner et al., USA 1988 [14]**	Cross-sectional	MMSE	11	11	<0.83	>0.83	6.3 ± 9.6	5.2 ± 2.1	t-test	.7
		self-care capacity (PGDRS)	11	11	<0.83	>0.83	13.2 ± 8.3	20.1 ± 1.5	t-test	.03
**Thomas et al., Germany 2008 [18]**	Cross-sectional	delirium (DI)	n = 15, cognitively unimpaired	n = 31, with dementia; n = 15, delirium with dementia	9.33 ± 4.44	11.03 ± 6.15; 12.25 ± 10.53	2.5 ± 0.7	6.2 ± 4.0; 8.7 ± 4.5	ANOVA and Duncan’s post-hoc-tests	< .02
		MMSE	n = 15, cognitively unimpaired	n = 31, with dementia; n = 15, delirium with dementia	9.33 ± 4.44	11.03 ± 6.15; 12.25 ± 10.53	28.8 ± 1.8	16.7 ± 7.5; 14.4 ± 6.0	ANOVA and Duncan’s post-hoc-tests	< .001
		IQCODE	n = 15, cognitively unimpaired	n = 31, with dementia; n = 15, delirium with dementia	9.33 ± 4.44	11.03 ± 6.15; 12.25 ± 10.53	3.1 ± 0.2	4.2 ± 0.6; 4.2 ± 0.7	ANOVA and Duncan’s post-hoc-tests	< .004
		SPMSQ	n = 15, cognitively unimpaired	n = 31, with dementia; n = 15, delirium with dementia	9.33 ± 4.44	11.03 ± 6.15; 12.25 ± 10.53	0.6 ± 0.9	4.7 ± 2.8; 6.4 ± 2.7	ANOVA and Duncan’s post-hoc-tests	< .001
		BI	n = 15, cognitively unimpaired	n = 31, with dementia; n = 15, delirium with dementia	9.33 ± 4.44	11.03 ± 6.15; 12.25 ± 10.53	62.5 ± 31.2	33.7 ± 24.3; 45.0 ± 18.1	ANOVA and Duncan’s post-hoc-tests	< .005
**Tune et al., USA 1993 [49]**	Cross-sectional	SAA level for delirious and non-delirious individuals	16	9	5.0 ± 2.41	7.09 ± 2.10	5.0 ± 2.41	7.09 ± 2.10	t-test	.045
**Case-control**										
**Mach Jr et al., USA 1995 [10]**	Case-control	MMSE	11	11	3.38 ± 2.49	6.05 ± 2.97	28 ± 1.3	26 ± 2.7	paired t-test	< .05
**Plaschke et al., Germany 2010 [60]**	Case-control	cognitive function (neuropsychological tests), cortisol and SAA correlation	23, low SAA group	7, high SAA group	3.3 ± 2.2	6.1 ± 3.9	not reported	not reported	ANOVA	N.S for both studies
**Plaschke et al., Germany 2013 [61]**	Case Control	IQCODE	n = 87, no POCD	n = 30, with POCD	4.5 ± 3.9	1.6 ± 1.7	86 (98.9)	28 (93.3)	χ^2^ test	.099
		subjective memory complaints	n = 87, no POCD	n = 30, with POCD	4.5 ± 3.9	1.6 ± 1.7	76 (87.4)	22 (73.3)	χ^2^ test	.073
**Thienhaus et al., USA 1990 [12]**	Case-control	MMSE	18	18	4.09 ± 4.83	6.66 ± 6.23	27.7 ± 1.2	27.9 ± 1.9	Paired t-test	N.S
		Digit Retention Span	18	18	4.09 ± 4.83	6.66 ± 6.23	6.2 ± 2.1	6.1 ± 1.7	Paired t-test	N.S
		word recognition	18	18	4.09 ± 4.83	6.66 ± 6.23	12.4 ± 5.3	14.0 ± 6.1	Paired t-test	N.S
		category retrieval	18	18	4.09 ± 4.83	6.66 ± 6.23	10.4 ± 4.0	10.5 ± 4.9	Paired t-test	N.S
		self-rated Memory	18	18	4.09 ± 4.83	6.66 ± 6.23	26.0 ± 38.4	27.2 ± 43.3	Paired t-test	N.S
**Prospective cohort**										
**Plaschke et al., Germany 2007 [44]**	Prospective, cohort	Correlation of SAA and cerebral spinal fluid anticholinergic activity.	n = 20	n = 17, with delirium	2.6 ± 2.3	2.8 ± 2.5	r = 0.861		Correlation between two numerical variables	>.05
**Remillard, Canada 1994 [43]**	Prospective cohort	MMSE	23, non-detectable SAA	8, detectable SAA	<1.8	>1.8	23.9 ± 3.7	25.2 ± 3.8	unpaired *t*-test	N.S
		SDM	23, non-detectable SAA	8, detectable SAA	<1.8	>1.8	15.2 ± 6.4	24.2 ± 8.7	unpaired *t*-test	< .001
**Rossi et al., Switzerland 2014 [45]**	Prospective cohort	MMSE	n = 38, no POCD	n = 32, with POCD	0.97 (.65, 1.83)	1.32 (.68, 2.59)	29 (28, 30)	27.5 (25, 29)	χ^2^ test	.004
		CERAD	n = 38, no POCD	n = 32, with POCD	0.97 (.65, 1.83)	1.32 (.68, 2.59)	99 (93, 105)	93 (85, 101)	*t*-test	.007
**Tune et al., USA 1981 [42]**	Prospective cohort	Correlation between delirium (MMSE) and SAA level.	19 controls	10 patients with delirium	Not clearly mentioned, but a SAA level >1.5 pmol is defined as high-SAA		r = -0.83		Correlation between two numerical variables	< .001
**Watne et al., Norway & UK 2014 [16]**	Prospective cohort	IQCODE	32, no delirium (Edinburg)	20, with delirium	1.62 (0.81–2.45) (IQR)	1.35 (0.76–2.46) (IQR)	0	5	Mann-Whitney test/ Chi-square test	.01
		Katz ADL	32, no delirium (Edinburg)	20, with delirium	1.62 (0.81–2.45) (IQR)	1.35 (0.76–2.46) (IQR)	31	16	Mann-Whitney test/ Chi-square test	.07
		IQCODE	44, no delirium (Oslo)	52, with delirium	6.08 (4.08–9.86) (IQR)	7.02 (4.24–9.73) (IQR)	10	42	Mann-Whitney test/ Chi-square test	< .001
		Barthel ADL	44, no delirium (Oslo)	52, with delirium	6.08 (4.08–9.86) (IQR)	7.02 (4.24–9.73) (IQR)	29	11	Mann-Whitney test/ Chi-square test	< .001

**Fig 2:**
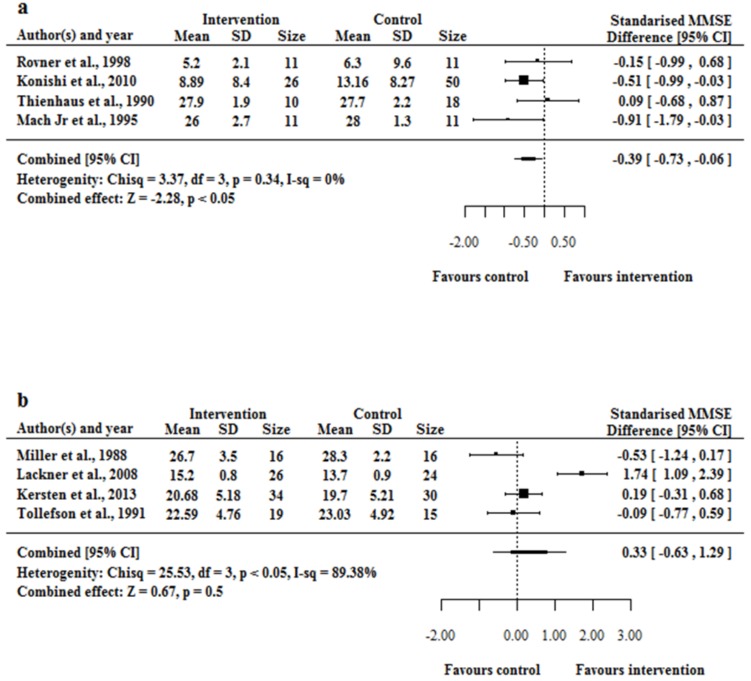
**(a and b)** Forest plot of standardised mean difference between increased SAA and a reduced MMSE score (positive favours MMSE reduction). Forest plot, using data from (a) 4 observational studies and (b) 4 randomised controlled trails following interventions that increase SAA and a decline in MMSE score. A) The result support SAA lowers the MMSE score (p < 0.05) for observational studies. B) The result did not support a conclusion that SAA lowers the MMSE score (p = 0.5) for RCTs.

Data from 4 cross-sectional and case-control studies [10, 12, 14, 55] were pooled for a meta-analysis. An initial random-effect modelling included all 5 studies (data not shown). The study by Hori et al. [57] was excluded from the analysis, as the funnel plot analysis revealed this study as a potential outlier that could bias the pooled estimate and the MMSE score mentioned in their article was adopted from Konishi et al. [55]. Pooled data from 4 observational studies using random effect modelling showed elevated SAA was associated with reduced cognitive performance (*I*^2^ = 0.00%, *H*^2^ = 3.37 and *p*-value = 0.34) (Fig 2A). The funnel-plot illustrates no outliers and excludes large-study bias (see S1 Fig).

Four RCTs met our inclusion criteria. One study that reported median MMSE scores with interquartile ranges was excluded from the analyses [41]. In the meta-analysis of RCT studies, the heterogeneity is statistically significant. A potential outlier [40] from the funnel plot analysis (see S2 Fig) was noted as the confidence interval does not overlap at all with the rest. After excluding the study by Lackner et al., the heterogeneity is reduced greatly and no longer statistically significant. Regardless of whether the study by Lackner et al. [40] is included or not, the conclusion remains unchanged (i.e. an increase in SAA level does not change MMSE score) (S3 Fig). In the final analyses, data from 4 RCTs were pooled and meta-analysed using random-effect modelling [11, 13, 40]. In Tollefson’s work [13], the treatment was designed to reduce SAA, so in random effects model, we considered ‘non-treatment’ as the intervention that increases SAA and the standardised effect size was relative to the post-treatment MMSE scores. The pooled data from 4 RCTs using random effect modelling found no association between elevated SAA and cognitive performance (*I*^2^ = 89.38%, *H*^2^ = 25.53 and *p*-value = <0.05) (Fig 2B).

A sensitivity analysis for the combined standardised MMSE differences was performed to verify that the observed effect size was not influenced by a particular study. This was accomplished by dropping out one study at a time and the standardised MMSE differences recalculated with confidence intervals. For the observational studies, the result favours the trend that an increase in SAA level lowers the MMSE score, but the changes were not always statistically significant. The study by Konishi et al. [55] demonstrated the adverse effect of high-SAA on MMSE scores, and their study size is much larger than the rest. This may have biased the result that an increase in SAA level changed MMSE scores. Finding from the RCT meta-analysis remained unchanged regardless of which study was excluded. The summary of sensitivity analysis findings is reported in S4 Table.

### Quality assessment

The qualities of the included RCT studies were critically appraised using the Cochrane risk assessment tool, highlighted in S5 Table. Two studies [11, 13] did not report the randomisation process, and there was general lack of adequate blinding, between participants and health professionals, and between outcomes and assessors.

The quality of the included case-control and cross-sectional studies were critically appraised using Newcastle Ottawa scale and the assessments are shown in S6 and S7 Tables. The qualities of included individuals in these studies were adequate, but in some of them, potential confounders were not discussed that may have lowered the reliability of the outcomes.

## Discussion

To our knowledge, this is the first systematic review that conducted a meta-analysis to quantify the association between SAA and adverse outcomes in older people.

The evidence from pooled analyses from 4 RCTs failed to confirm an association between SAA and impaired cognitive performance (Fig 2B). However, evidence from 4 observational studies shows an association between SAA and cognitive performance (Fig 2A). There were several methodological limitations that hindered inclusion of studies into the meta-analysis. Several studies failed to blind the participant and the health professional [41], and blinding of outcomes and the assessments were not undertaken [13]. The observed relationship between the intervention and change in MMSE scores may be confounded by participant selection and different outcome assessments. Included studies considered reporting of MMSE scores before and after the intervention, but very limited details were provided with respect to participant’s follow-up and information on when MMSE measurements were undertaken.

### SAA and cognitive outcomes

The MMSE scale was widely employed to measure cognitive performance followed by the Informant Questionnaire on Cognitive Decline in the Elderly (IQCODE), Consortium to Establish a Registry for Alzheimer Disease (CERAD), Confusion Assessment Method (CAM). MMSE is one of the globally accepted tools for measuring cognitive impairment in older people, however a recent systematic review highlighted that the sensitivity of MMSE for measuring cognitive improvements may be low in nursing home residents and MMSE as a tool has not been evaluated for measuring changes caused by drug-induced cognitive impairment [4]. In addition, studies reported that MMSE may not be an optimal method to detect mild drug-induced cognitive changes in older people [11, 17, 64].

The studies included in this review confirm a consistent correlation between higher SAA and worsening cognitive performance [7, 53, 60, 65]. However, negative association between cognitive performance and SAA were identified as well. A study by Remillard [43] reported similar findings to those of Rovner et al. [14] regarding negative associations between SAA and MMSE scores. Thomas and colleagues [18] failed to show a positive correlation between higher SAA and cognitive function using the cerebral cholinergic function measured with electroencephalography. Findings from this study suggests a poor correlation between peripheral SAA levels and cognitive effects.

The qualitative and quantitative findings from this review showed that SAA is associated with an increased risk of delirium, assessed using the Diagnostic and Statistical Manual of Mental Disorders (DSM) criteria [10, 46, 49] and also from other derivatives such as the CAM [40, 47, 53], CAM-ICU [44] for critically-ill individuals, delirium index (DI) [18] and Saskatoon Delirium Checklist (SDC) [11]. A study by Plaschke et al. [44] evaluated the correlation of SAA with delirium in critically-ill patients. However, the study findings showed higher SAA value in the delirious cohort was not correlated with a risk of developing delirium.

### SAA and functional outcomes

Findings from a recent cross-sectional study [18] conducted in Germany reported a moderate correlation of SAA with functional outcomes, indicating an inverse relationship of the anticholinergic burden on functional capacity in non-delirious individuals and cognitively unimpaired however lack of correlation was reported in individuals with dementia and delirium indicating the role of additional mechanisms that leads to functional deficits. Another cross-sectional study by Nebes et al. [23] reported higher SAA was associated with slowing of psychomotor tasks including gait speed and simple manual response times. A cross-sectional study involving 67 older medical inpatients failed to find an association between SAA levels and ADLs among older people [51]. Methodological limitations hampered the completion of meta-analyses of data from studies that examined a relationship between SAA and functional outcomes.

### Variations in SAA levels

A RCT study conducted by Miller et al. [11] in presurgical older patients found a huge variation in mean SAA values ranging from 9.1 ± 17.7 pmol/ml atropine equivalents and increased to 121.1 ± 85.5 pmol/ml atropine equivalents after administration of scopolamine. A cross-sectional study [17] reported detectable SAA of 2.27 pmol/ml in the absence of anticholinergic medicines. Another study conducted by Tune et al. reported absolute mean SAA level greater than 7.5 pmol/mL in surgical patients experiencing delirium, compared with less than 7.5 pmol/mL in surgical patients without delirium [42]. SAA levels associated with delirium or cognitive decline varied considerably in these studies [20]. A detailed summary of variations in SAA measurement is highlighted in Table 2.

SAA is an important biomarker to understand cognitive impairment, but has several limitations. Findings of this systematic review failed to confirm a threshold level of SAA that predicts delirium or cognitive decline. SAA measures anticholinergic activity in the blood rather than in the central nervous system and there is a poor correlation between peripheral anticholinergic activity measured by SAA and central nervous system effects [25, 37, 66]. Endogenous substances in addition to anticholinergic medicines and their metabolites have also shown to affect SAA measurements [21]. The standardisation of bioassay reported in the studies is also uncertain [20] and the reasons could be intra-laboratory variability in bioassay methods and heterogeneity of study populations [25]. The summary of potential limitations of SAA measurement is depicted in S8 Table.

### Strengths and limitations

This systematic review was comprehensive in that the electronic search conducted in 4 different databases endeavoured to identify all potential studies that met our eligibility criteria. We used the PICO (population, intervention, comparison and outcome) criteria to frame research questions. The population mean age was set to 55 years to capture potential studies, especially from the USA, to be included in this review. The review explicitly looked into SAA measurement and its association with adverse outcomes. The objectives were clearly stated and the search methodology including the citation analysis were robust. A systematic approach was used to synthesise and characterise the findings of this review followed by a meta-analysis.

The exact relationship between SAA and cognitive functions in older people remains unclear [9]. Only a small number of medications were assessed using SAA method and the metabolites of these medications remain unexamined [8, 9]. The variation in study methodologies prevented several case-control studies, longitudinal studies and prospective cohorts to be excluded from the meta-analyses. The inconsistent measure of cognitive and functional outcomes reported in the studies limited the meta-analysis. The review found associations between SAA and cognitive adverse outcomes, but not with functional outcomes. Larger well designed experimental studies are needed to confirm these associations. Notwithstanding, there are several technical concerns for the utility of SAA [19,20] and further discussions are needed to determine the utility of SAA in clinical practice.

## Conclusions

This systematic review and meta-analysis examined studies that used SAA as a method to quantify anticholinergic burden and examined associations with adverse outcomes in older people. The complexity of assessing anticholinergic burden using the SAA method limits its widespread acceptance as a biomarker to assess anticholinergic effects. The evidence from pooled analyses from 4 RCTs failed to confirm an association between SAA and impaired cognitive performance. Though, evidence from 4 observational studies shows an association between SAA and cognitive performance. SAA measured by receptor bioassay is flawed and its use in older people with multimorbidity and polypharmacy is questionable. In conclusion, SAA has a number of limitations as a biomarker for predicting cognitive impairment in older people.

## Supporting Information

S1 Fig Funnel plot of 4 observational studies included in meta-analysis examining the relationship between high SAA and cognitive outcome (MMSE) in older people. Funnel plot shows the validity of the meta-analysis on the cross-sectional and case-control studies. All data falls within the allowable region of the funnel plot, indicating that the analysis does not involve outliers that are overrepresented in the analysis
(TIF)

S2 Fig Funnel plot of 3 RCT studies included in meta-analysis examining the relationship between high SAA and cognitive outcome (MMSE) in older people. Funnel plot shows the validity of the meta-analysis on the RCT studies. All data falls within the allowable region of the funnel plot, indicating that the analysis does not involve outliers that are overrepresented in the analysis.
(TIF)

S3 Fig Forest plot of standardised mean difference between increased SAA and a reduced MMSE score (positive favours MMSE reduction). The result did not support a conclusion that SAA lowers the MMSE score for RCTs.
(TIF)

S1 Table MEDLINE search strategy. (DOCX)

S2 Table Summary of excluded studies from the systematic review. (DOCX)

S3 Table Summary of heterogeneity information of the included studies examining association between SAA and delirium. (DOCX)

S4 Table Summary of sensitivity analysis for both observational studies and RCTs. (DOCX)

S5 Table The Cochrane Risk of Bias tool results for included RCTs. (DOCX)

S6 Table The Newcastle-Ottawa scale risk of bias assessment for cohort studies. (DOCX)

S7 Table The Newcastle-Ottawa scale risk of bias assessment for included case-control study. (DOCX)

S8 Table Potential limitations of serum anticholinergic activity (SAA) measurement. (DOCX)

S9 Table The PRISMA checklist for systematic review. (DOCX)
