# Supplementary figures and images for: Serum Anticholinergic Activity and Cognitive and Functional Adverse Outcomes in Older People: A Systematic Review and Meta-Analysis of the Literature
Source: PLoS One. 2016 Mar 21;11(3):e0151084. doi: 10.1371/journal.pone.0151084 (PMC4801377; doi:10.1371/journal.pone.0151084)

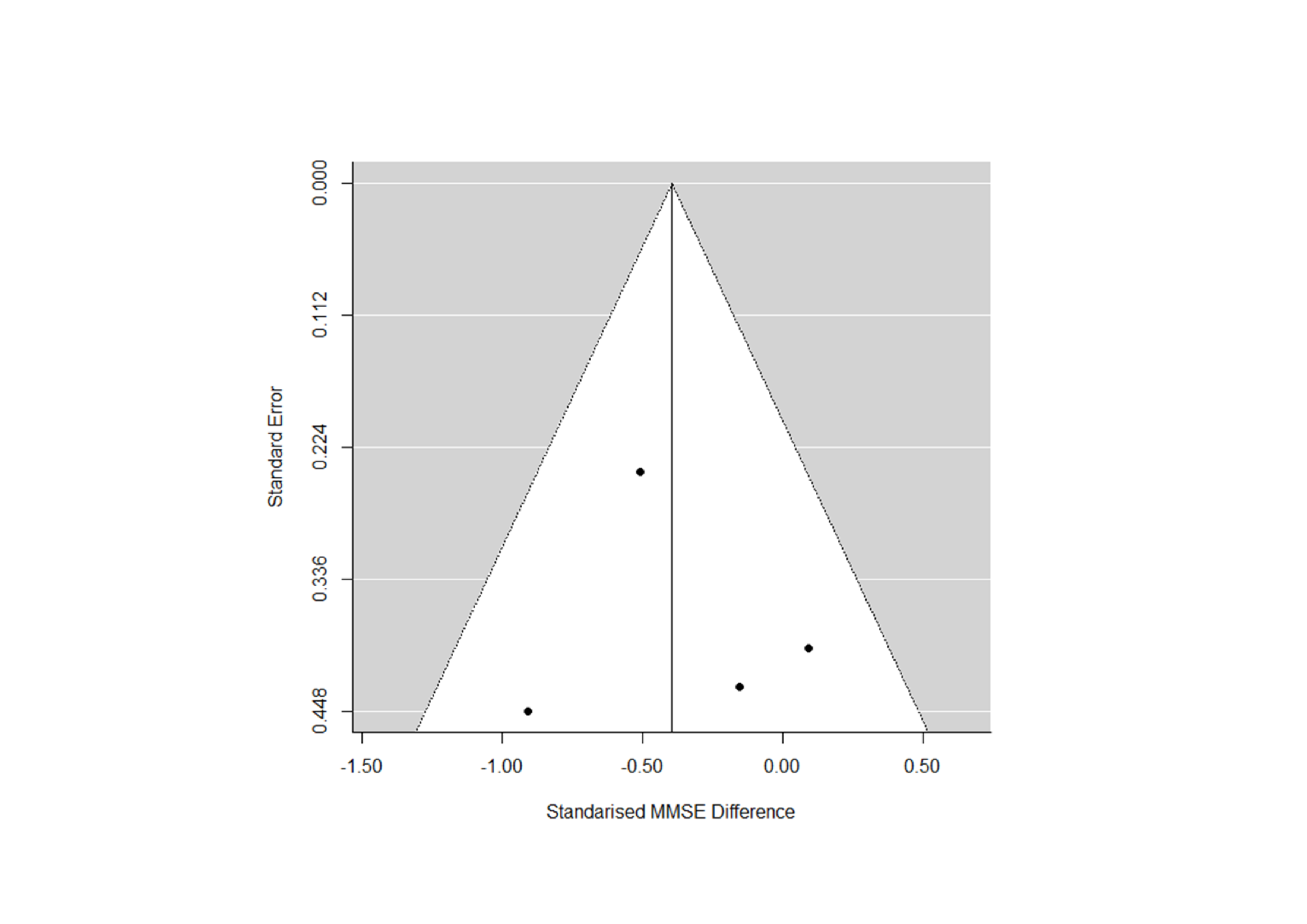

Supplement: S1 Fig — Funnel plot shows the validity of the meta-analysis on the cross-sectional and case-control studies. All data falls within the allowable region of the funnel plot, indicating that the analysis does not involve outliers that are overrepresented in the analysis (TIF) [file pone.0151084.s001.tif]

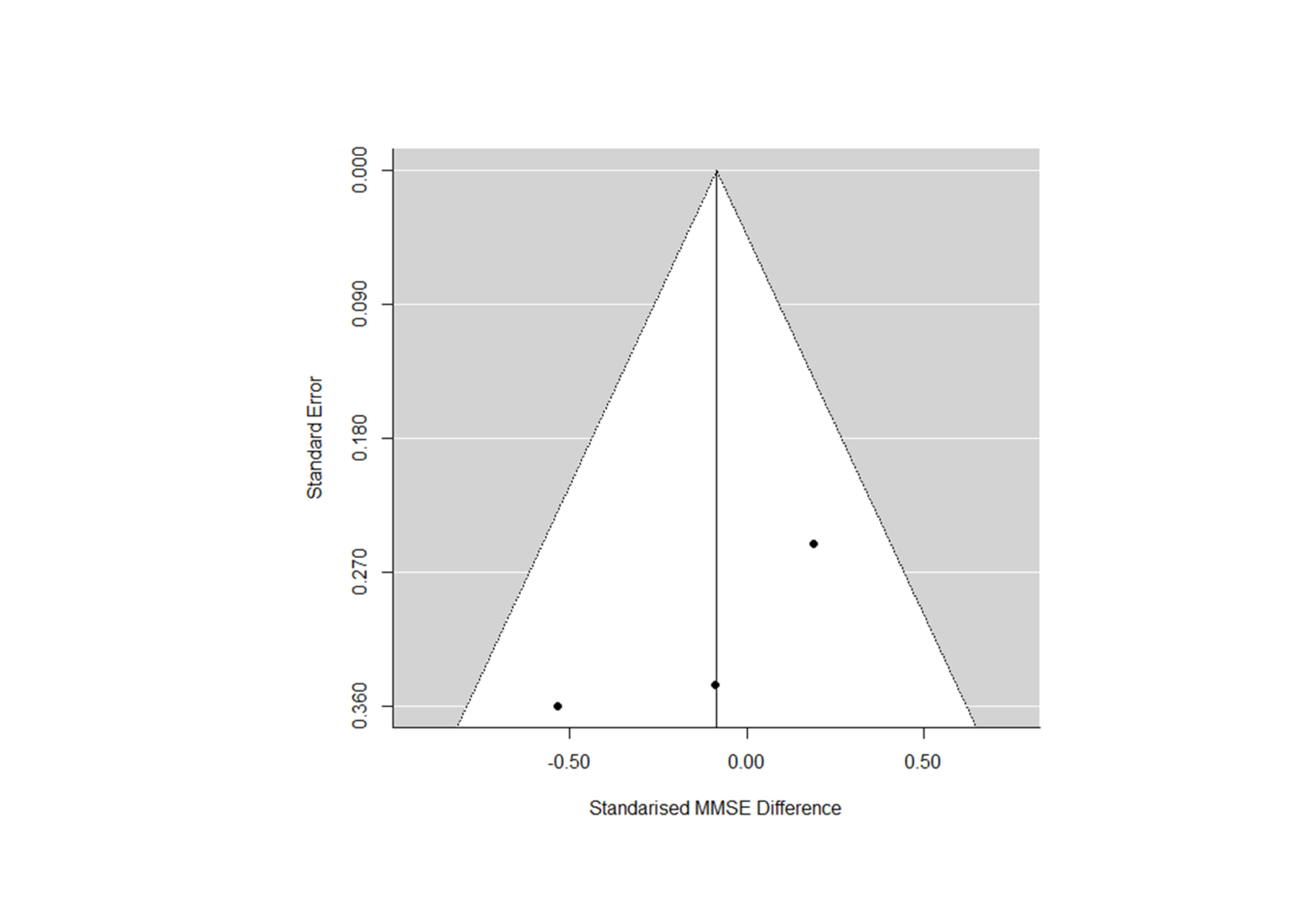

Supplement: S2 Fig — Funnel plot shows the validity of the meta-analysis on the RCT studies. All data falls within the allowable region of the funnel plot, indicating that the analysis does not involve outliers that are overrepresented in the analysis. (TIF) [file pone.0151084.s002.tif]

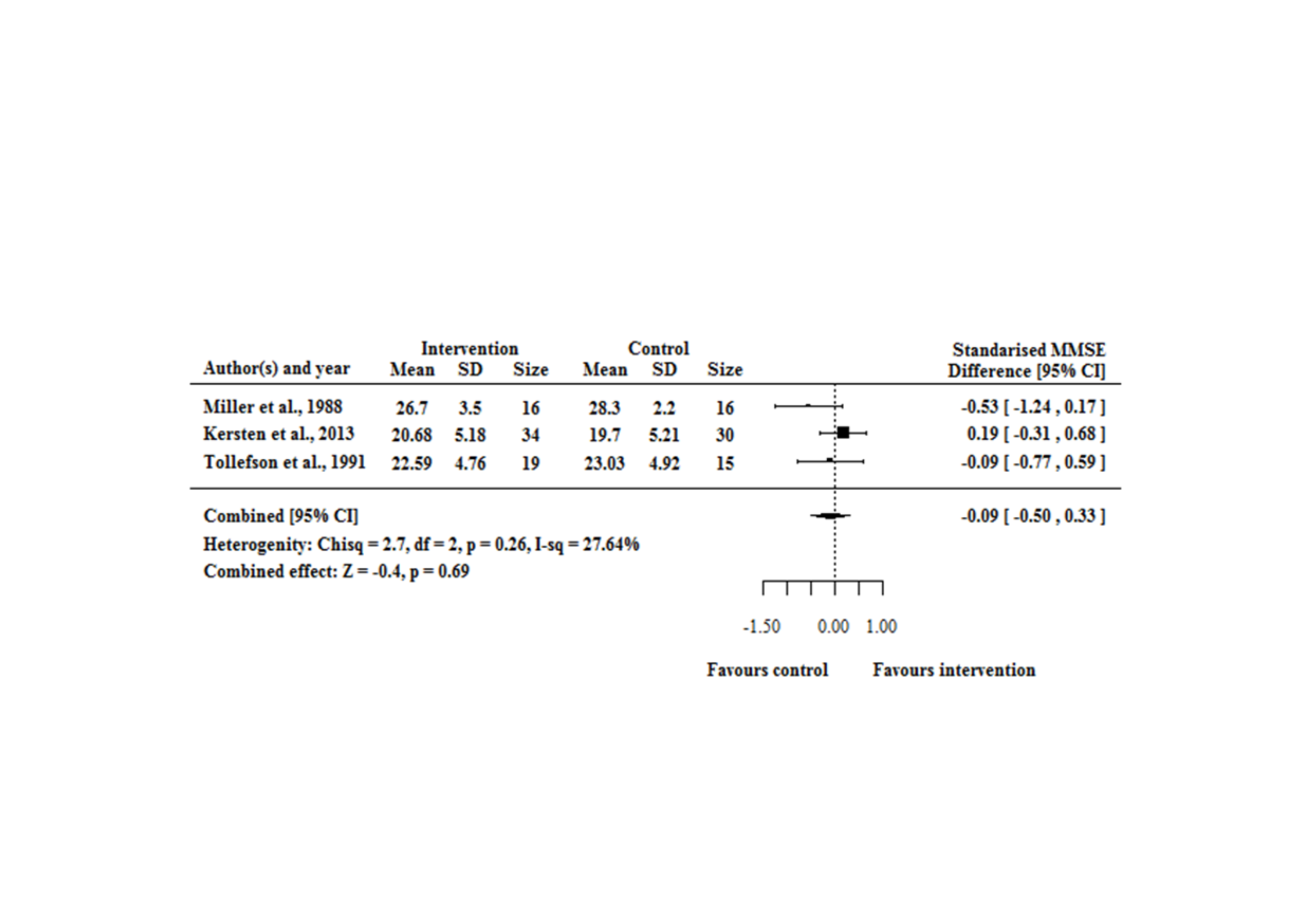

Supplement: S3 Fig — The result did not support a conclusion that SAA lowers the MMSE score for RCTs. (TIF) [file pone.0151084.s003.tif]
