# Supplementary material for: Serum Anticholinergic Activity and Cognitive and Functional Adverse Outcomes in Older People: A Systematic Review and Meta-Analysis of the Literature
Source: PLoS One. 2016 Mar 21;11(3):e0151084. doi: 10.1371/journal.pone.0151084 (PMC4801377; doi:10.1371/journal.pone.0151084)
Supplement: S1 Table — (DOCX) [file pone.0151084.s004.docx]

**S1 Table. MEDLINE search strategy**

| **Source** | **Search strategy: keyword/MeSH** |
| --- | --- |
| MEDLINE (Ovid SP) 1946 to September 2014 | 1. anticholinergic activity.mp. or anticholinergic burden.mp. or anticholinergic load.mp. |
|  | 1. radioreceptor assay.mp. or exp Radioligand Assay/ or antimuscarinic activity.mp. or serum a#.mp. or serum anticholinergic activity.mp. or SAA.ab |
|  | 1. receptors, muscarinic/ or exp receptor, muscarinic m1/ or exp receptor, muscarinic m3/ |
|  | 1. or/1-3 |
|  | 1. Cognition Disorders/ or Cognition/ or cogniti*.mp. |
|  | 1. Delirium/ or exp Delirium, Dementia, Amnestic, Cognitive Disorders/ |
|  | 1. Memory/ or exp Memory, Long-Term/ or exp Memory, Short-Term/ |
|  | 1. exp Dementia/ or exp "Quality of Life"/ |
|  | 1. physical func*.mp. or physica#.mp. or physical activ#.mp. or functiona*.mp. or adverse outcom#.mp. |
|  | 1. or/5-9 |
|  | 1. exp Aged/ or exp "Aged, 80 and over"/ or elder*.mp. or frail.mp. or geriatric*.mp. or seniors.mp. or “old#.mp. |
|  | 1. 4 and 10 and 11 |
|  | 1. limit 12 to (English language and humans) |
