# Supplementary material for: Serum Anticholinergic Activity and Cognitive and Functional Adverse Outcomes in Older People: A Systematic Review and Meta-Analysis of the Literature
Source: PLoS One. 2016 Mar 21;11(3):e0151084. doi: 10.1371/journal.pone.0151084 (PMC4801377; doi:10.1371/journal.pone.0151084)
Supplement: S2 Table — (DOCX) [file pone.0151084.s005.docx]

**S2 Table. Summary of excluded studies from the systematic review**

| **Studies excluded** | **Study design** | **Study setting/ participants** | **Mean (SD) age (years)** | **Study duration** | **Reason for exclusion** |
| --- | --- | --- | --- | --- | --- |
| Ancelin et al, France 2006 [36] | Longitudinal cohort | Community, N = 372 | > 60 | 8 years | Anticholinergic burden was quantified based on scores given by the rating scale |
| Bottiggi et al, 2006 [27] | Longitudinal retrospective cohort | Cognitive test data, N = 592 | 72.6 (7) | 6 years | This study did not consider SAA method |
| Brecht et al, Germany 2007 [32] | Prospective study | Hospital, N = 26 | aged 25-80 (study 1)  aged 25-45 (study 2) | unclear | Mean age of the participants were below 55 years (study 2) and the outcome measures were not studied as per the inclusion criteria |
| Carnahan et al, USA 2006 [35] | Cross-sectional | Long-term care residents, N = 279 | 86 (7) | 1 month | Study did not consider assessment of adverse outcome. It only measured rating for individual drugs based on SAA and expert opinion |
| Landi et al, Italy 2007 [28] | Prospective cohort | Community living, N = 364 | 85.9 (4.8) | 9 months | Study used SAA values previously demonstrated in literature |
| Mondimore et al, USA 1983 [30] | Cross-sectional | post-ECT inpatients, N = 20 | 49 (17) | unclear | Mean age of the participants were below 55 years |
| Plaschke et al, Germany 2007 [37] | Cohort study | Inpatients, N = 15 | 70.4 (6) | unclear | Study did not consider assessment of adverse outcome |
| Richardson et al, Canada 1994 [31] | RCT | Psychiatric clinics, N = 37 | aged 18-70 | 3 weeks | Mean age of the participants were below 55 years |
| Tune et al, USA 1982 [29] | Cross-sectional | Outpatients, N = 24 | average age 35.7 | unclear | Mean age of the participants were below 55 years |
| Tune et al, USA 1992 [34] | Prospective study | unclear | not defined | not defined | Study measured only the effects of 25 commonly prescribed anticholinergic medicines in the elderly using radioreceptor assay and not described any associations with adverse outcomes |
| Vinogradov et al, USA 2009 [33] | RCT | Patients with schizophrenia, N = 49 | 43.86 (10.29) | 10 weeks | Mean age of the participants were below 55 years |

RCT = Randomised controlled trial; SAA = Serum Anticholinergic Activity
