# Supplementary material for: Serum Anticholinergic Activity and Cognitive and Functional Adverse Outcomes in Older People: A Systematic Review and Meta-Analysis of the Literature
Source: PLoS One. 2016 Mar 21;11(3):e0151084. doi: 10.1371/journal.pone.0151084 (PMC4801377; doi:10.1371/journal.pone.0151084)
Supplement: S3 Table — (DOCX) [file pone.0151084.s006.docx]

**S3 Table. Summary of heterogeneity information of the included studies examining association between SAA and delirium**

| **Studies used SAA** | **Study design** | **Study setting / participants** | **Statistical test** | **Adverse outcome(s) studied** | **Significant association** | **Remarks** |
| --- | --- | --- | --- | --- | --- | --- |
| **RCT** | | | | | | |
| Lackner et al, USA 2008 [40] | RCT, double-blinded | nursing home | Correlation analysis | delirium (CAM) | – | In this short-term trial, delirium was not detected at any time point. The presence or absence of delirium as determined according to the CAM was used as a secondary outcome measure. |
| Miller et al, Canada 1988 [11] | RCT, double blinded | hospitalised pts | ANCOVA | delirium (SDC) | + | The study used SDC checklist to detect the cognitive changes produced by very low levels of anticholinergic activity. |
| **Longitudinal cohort** | | | | | | |
| Golinger et al, USA 1987 [46] | Longitudinal cohort | surgical ICU pts | t-test | Delirium (DSM) | + | No numerical values reported. |
| van Munster et al, Netherlands 2012 [47] | Longitudinal study | hospitalised pts | Mixed-model regression | delirium (CAM) | – | This study assessed the possible association between SAA and delirium in elderly patients with acute hip fracture and subsequent surgical repair.  The close occurrence of fracture and surgery as well as once-daily delirium assessments might have missed some fluctuating states. |
| **Cross-sectional** | | | | | | |
| Mussi et al, Italy 1999 [53] | Cross-sectional | hospitalised pts | t-test | delirium (CAM) with elevated SAA | + | Patients were divided into two groups, depending on the presence or the absence of delirium. |
| Thomas et al, Germany 2008 [18] | Cross-sectional | hospitalised pts | ANOVA and Duncan’s post-hoc-tests | delirium (DI) | + | Delirium was evaluated using delirium index method |
| Tune et al, USA 1993 [49] | Cross-sectional | surgical ICU pts | t-test | Delirium (DSM) | + | No numerical values reported. |
| **Case-control** | | | | | | |
| Mach Jr et al, USA 1995 [10] | Case-control | hospitalised pts | t-test | Delirium (DSM) | + | Delirium measured for selection of participants. |
| **Prospective cohort** | | | | | | |
| Plaschke et al, Germany 2007 [44] | Prospective, cohort | ICU pts | Correlation between two numerical variables | delirium using electroencephalographic data (CAM-ICU) | – | CAM-ICU was used to assess delirium. |
| Tune et al, USA 1981 [42] | Prospective cohort | postcardiotomy patients | Correlation | delirium | + | SAA level was not mentioned.  The study describe diagnosis of delirium; ‘when there was an acute change in the patient’s mental state characterised by alteration in the level of consciousness, by disorientation in time and place, and by cognitive impairment.’ |

SAA = serum anticholinergic activity; RCT = randomised controlled trial; pts = patients; ICU = intensive care unit; SDC = Saskatoon delirium checklist; CAM = confusion assessment method; DI = delirium index; ANCOVA = analysis of covariance; ANOVA = analysis of variance; DSM = Diagnostic and Statistical Manual of Mental Disorders.
