# Supplementary material for: Serum Anticholinergic Activity and Cognitive and Functional Adverse Outcomes in Older People: A Systematic Review and Meta-Analysis of the Literature
Source: PLoS One. 2016 Mar 21;11(3):e0151084. doi: 10.1371/journal.pone.0151084 (PMC4801377; doi:10.1371/journal.pone.0151084)
Supplement: S4 Table — (DOCX) [file pone.0151084.s007.docx]

**S4 Table. Summary of sensitivity analysis for both observational studies and RCTs.**

| **Study excluded** | **Combined Standardized MMSE difference [95% CI]** | **Significance** |
| --- | --- | --- |
| **Case-control** | | |
| Rovner et al., 1998 | -0.44[-0.88,0.01] | p=0.05 |
| Konishi et al., 2010 | -0.29[-0.87,0.28] | p=0.23 |
| Thienhaus et al., 1990 | -0.51[-0.89,-0.13] | P<0.05 |
| Mach Jr et al., 1995 | -0.29[-0.68,0.1] | p=0.15 |
| **RCTs** | | |
| Miller et al., 1998 | 0.09[-0.31,0.49] | p=0.65 |
| Kersten et al., 2013 | -0.30[-0.79,0.19] | p=0.23 |
| Tollefson et al., 1991 | -0.13[-0.83,0.57] | p=0.72 |

RCTs = Randomised controlled trials
