# Supplementary material for: Serum Anticholinergic Activity and Cognitive and Functional Adverse Outcomes in Older People: A Systematic Review and Meta-Analysis of the Literature
Source: PLoS One. 2016 Mar 21;11(3):e0151084. doi: 10.1371/journal.pone.0151084 (PMC4801377; doi:10.1371/journal.pone.0151084)
Supplement: S5 Table — (DOCX) [file pone.0151084.s008.docx]

**S5 Table. The Cochrane Risk of Bias tool results for included RCTs.**

| **Study** | **Study design** | **Randomisation sequence generation** | **Allocation concealment** | **Blinding of participants and personnel** | **Blinding outcome assessors** | **Loss to follow up (incomplete data)** | **Selective reporting** |
| --- | --- | --- | --- | --- | --- | --- | --- |
| Kersten et al, Norway 2013 [41] | RCT, single-blinded | 🗸 | X | X | ? | X | 🗸 |
| Lackner et al, USA 2008 [40] | RCT, double-blinded | 🗸 | X | 🗸 | 🗸 | X | 🗸 |
| Miller et al, Canada 1988 [11] | RCT, double blinded | ? | X | 🗸 | ? | X | 🗸 |
| Tollefson et al, USA 1991 [13] | RCT repeated measured | ? | X | ? | X | X | 🗸 |

X = inadequate; 🗸 = adequate; ? = unclear

RCT = Randomised controlled trial
