# Supplementary material for: Serum Anticholinergic Activity and Cognitive and Functional Adverse Outcomes in Older People: A Systematic Review and Meta-Analysis of the Literature
Source: PLoS One. 2016 Mar 21;11(3):e0151084. doi: 10.1371/journal.pone.0151084 (PMC4801377; doi:10.1371/journal.pone.0151084)
Supplement: S6 Table — (DOCX) [file pone.0151084.s009.docx]

**S6 Table. The Newcastle-Ottawa scale risk of bias assessment for included cohort studies**

| **Study** | **Study design** | **Adverse outcome(s)** | **Selection (Total 4)** | **Comparability (Total 2)** | **Outcome (Total 3)** |
| --- | --- | --- | --- | --- | --- |
| Golinger et al, USA 1987 [46] | Longitudinal cohort | cognitive | ✯✯ | ✯ | ✯ |
| Kashyap et al, Canada 2014 [48] | Longitudinal cohort | cognitive | ✯✯✯✯ | ✯✯ | ✯✯✯ |
| van Munster et al, Netherlands 2012 [47] | Longitudinal study | cognitive | ✯✯✯ | ✯✯ | ✯✯✯ |
| Chew et al, USA 2005 [8] | Cross-sectional | cognitive | ✯✯✯ | ✯✯ | ✯✯✯ |
| Flacker et al, USA 1998 [51] | Cross-sectional | functional | ✯✯✯ | ✯✯ | ✯✯✯ |
| Flacker et al, USA 1999 [52] | Cross-sectional | cognitive | ✯✯✯ | ✯✯ | ✯✯✯ |
| Hori et al, Japan 2011 [57] | Cross-sectional | cognitive, functional | ✯✯✯ | ✯✯ | ✯✯✯ |
| Kersten et al, Norway 2013 [58] | Cross-sectional | cognitive, functional | ✯✯✯✯ | ✯✯ | ✯✯✯ |
| Konishi et al, Japan 2010 [55] | Cross-sectional | cognitive, functional | ✯✯✯ | ✯✯ | ✯✯✯ |
| Lampela et al, Finland 2013 [17] | Cross-sectional | cognitive, functional | ✯✯✯✯ | ✯✯ | ✯✯✯ |
| Mangoni et al, Netherlands 2013 [59] | Cross-sectional | cognitive, functional | ✯✯✯ | ✯✯ | ✯✯✯ |
| Mulsant et al, USA 2003 [7] | Cross-sectional | cognitive | ✯✯✯✯ | ✯✯ | ✯✯✯ |
| Mussi et al, Italy 1999 [53] | Cross-sectional | cognitive | ✯✯✯ | ✯✯ | ✯✯✯ |
| Nebes et al, USA 1997 [50] | Cross-sectional | cognitive | ✯✯✯ | ✯✯ | ✯✯✯ |
| Nebes et al, USA 2005 [54] | Cross-sectional | cognitive | ✯✯✯✯ | ✯ | ✯ |
| Nebes et al, USA 2007 [23] | Cross-sectional | functional | ✯✯✯✯ | ✯✯ | ✯ |
| Nebes et al, USA 2011 [56] | Cross-sectional | cognitive | ✯✯✯ | ✯✯ | ✯ |
| Rovner et al, USA 1988 [14] | Cross-sectional | cognitive, functional | ✯✯✯ | ✯ | ✯ |
| Thomas et al, Germany 2008 [18] | Cross-sectional | cognitive, functional | ✯✯✯ | ✯ | ✯✯✯ |
| Tune et al, USA 1993 [49] | Cross-sectional | cognitive | ✯✯✯ | ✯ | ✯ |
| Plaschke et al, Germany 2007 [44] | Prospective, cohort | cognitive | ✯✯✯ | ✯✯ | ✯✯ |
| Remillard, Canada 1994 [43] | Prospective cohort | cognitive | ✯✯✯✯ | ✯ | ✯ |
| Rossi et al, Switzerland 2014 [45] | Prospective cohort | cognitive | ✯✯✯ | ✯✯ | ✯✯ |
| Tune et al, USA 1981 [42] | Prospective cohort | cognitive | ✯✯ | ✯ | ✯ |
| Watne et al, Norway & UK 2014 [16] | Prospective cohort | cognitive, functional | ✯✯✯✯ | ✯✯ | ✯✯✯ |
