# Supplementary material for: Serum Anticholinergic Activity and Cognitive and Functional Adverse Outcomes in Older People: A Systematic Review and Meta-Analysis of the Literature
Source: PLoS One. 2016 Mar 21;11(3):e0151084. doi: 10.1371/journal.pone.0151084 (PMC4801377; doi:10.1371/journal.pone.0151084)
Supplement: S7 Table — (DOCX) [file pone.0151084.s010.docx]

**S7 Table. The Newcastle-Ottawa scale risk of bias assessment for included Case-Control studies**

| **Study** | **Study design** | **Adverse outcome(s)** | **Selection (Total 4)** | **Comparability (Total 2)** | **Exposure (Total 3)** |
| --- | --- | --- | --- | --- | --- |
| Mach Jr et al, USA 1995 [10] | Case-control | cognitive | ✯✯✯✯ | ✯✯ | ✯✯✯ |
| Plaschke et al, Germany 2010 [60] | Case-control | cognitive | ✯✯✯✯ | ✯✯ | ✯✯✯ |
| Plaschke et al, Germany 2013 [61] | Case Control | cognitive | ✯✯✯✯ | ✯✯ | ✯✯✯ |
| Thienhaus et al, USA 1990 [12] | Case-control | cognitive | ✯✯✯✯ | ✯ | ✯✯ |
