# Supplementary material for: Serum Anticholinergic Activity and Cognitive and Functional Adverse Outcomes in Older People: A Systematic Review and Meta-Analysis of the Literature
Source: PLoS One. 2016 Mar 21;11(3):e0151084. doi: 10.1371/journal.pone.0151084 (PMC4801377; doi:10.1371/journal.pone.0151084)
Supplement: S8 Table — (DOCX) [file pone.0151084.s011.docx]

**S8 Table. Potential limitations of serum anticholinergic activity (SAA) measurement**

| - The quantitative relationship between SAA and anticholinergic activity is poorly understood [15, 32, 33, 35] |
| --- |
| - SAA measures peripheral endogenous and exogenous anticholinergic activity, and levels do not correlate with central nervous system activity [18, 20, 21] |
| - Medicines that poorly cross the blood brain barrier such as quaternary ammonium compounds (e.g., propantheline), may contribute to SAA without exerting central nervous system effects, but they may still have peripheral antimuscarinic effects [37, 66] |
| - 3H-QNB (quinuclidinyl benzilate) is a non-specific muscarinic antagonist and it binds with all subtypes of muscarinic receptors, however it cannot be used to explain the affinity and specificity towards muscarinic receptor subtypes M1-M4. Receptor assays are designed to optimise the binding of antagonists but may also reflect binding of agonists and may not be a pure measure of anticholinergic activity [66] - The bioassay method is not standardised and the range of SAA varies from inter-laboratories and also by heterogeneity of study populations [25, 67] - The measurement of total SAA does not provide guidance on which anticholinergic medicine is to be discontinued [4] |
| - Measurement of SAA is an expensive procedure, invasive, not readily accessible and hard to interpret in clinical practice [20, 59, 66] |
| - Medical conditions and diseases that may affect the blood-brain-barrier permeability are mainly meningitis, brain abscess, Alzheimer’s disease, cerebral oedema, multiple sclerosis, epilepsy etc. [68-70] |
| - Plasma proteins might ‘‘trap’’ the muscarinic radioligand (3HQNB) or bind to the muscarinic receptors and thereby prohibit receptor docking of the ligand - Majority of the studies have used sonicated rat cerebrum in the in vitro bioassay and even though, all five muscarinic receptor subtypes are represented in the rat cerebrum, the amount of each receptor subtype might differ from the human cerebrum - The clinical use of SAA as a biomarker in older people is also limited by the individual variability in permeability across the blood-brain-barrier, this variability is amplified by different diseases and drugs that might interact with P-glycoprotein (PgP) and the influx/efflux of PgP-substrates to the brain |

**Additional References to the Supplementary Material S8 Table**

67. Collamati A, Martone AM, Poscia A, Brandi V, Celi M, Marzetti E, et al. Anticholinergic drugs and negative outcomes in the older population: from biological plausibility to clinical evidence. Aging Clin Exp Res. 2015. Epub 2015/05/02. doi: 10.1007/s40520-015-0359-7. PubMed PMID: 25930085.

68. Raza MW, Shad A, Pedler SJ, Karamat KA. Penetration and activity of antibiotics in brain abscess. J Coll Physicians Surg Pak. 2005;15(3):165-7. Epub 2005/04/06. doi: 03.2005/jcpsp. PubMed PMID: 15808097.

69. Zipser BD, Johanson CE, Gonzalez L, Berzin TM, Tavares R, Hulette CM, et al. Microvascular injury and blood-brain barrier leakage in Alzheimer's disease. Neurobiol Aging. 2007;28(7):977-86. Epub 2006/06/20. doi: 10.1016/j.neurobiolaging.2006.05.016. PubMed PMID: 16782234.

70. Waubant E. Biomarkers indicative of blood-brain barrier disruption in multiple sclerosis. Dis Markers. 2006;22(4):235-44. Epub 2006/11/25. PubMed PMID: 17124345; PubMed Central PMCID: PMCPmc3850823.
